# Methanol regulated yeast promoters: production vehicles and toolbox for synthetic biology

**DOI:** 10.1186/s12934-015-0387-1

**Published:** 2015-12-02

**Authors:** Brigitte Gasser, Matthias G. Steiger, Diethard Mattanovich

**Affiliations:** Department of Biotechnology, BOKU-University of Natural Resources and Life Sciences Vienna, Muthgasse 18, 1190 Vienna, Austria; Austrian Centre of Industrial Biotechnology (ACIB GmbH), Muthgasse 11, Vienna, Austria

**Keywords:** *Pichia pastoris*, *Komagataella*, Promoter, Induction, Synthetic biology, Protein production

## Abstract

Promoters are indispensable elements of a standardized parts collection for synthetic biology. Regulated promoters of a wide variety of well-defined induction ratios and expression strengths are highly interesting for many applications. Exemplarily, we discuss the application of published genome scale transcriptomics data for the primary selection of methanol inducible promoters of the yeast *Pichia pastoris* (*Komagataella* sp.). Such a promoter collection can serve as an excellent toolbox for cell and metabolic engineering, and for gene expression to produce heterologous proteins.

## Background

A major task of synthetic biology is the provision of standardized elements for rapid assembly of predictable recombinant gene expression cassettes [1, 2]. These elements include vectors, selection markers, and most importantly collections of regulatory elements like promoters, transcription terminators, secretory leaders and other signal sequences. Ideally, collections of these parts are cataloged in standardized, easy to assemble formats like BioBrick [3]. Promoters are indispensable parts for synthetic biology approaches [4] and are needed for different expression strength in order to balance the expression levels in a synthetic pathway [5]. There are a plethora of studies which characterize, e.g. constitutive promoters of different strength for *Escherichia coli* [6], *Aspergillus niger* [7] or *Pichia pastoris* [8]. Depending on the application it might be necessary to tightly control the promoter activity. Especially regulated promoters are often strictly host specific, so that they need to be identified, characterized and standardized for the host species of interest, as shown e.g. for *E. coli* [9].

### Methanol regulated promoters

Methylotrophic yeasts such as *P. pastoris* (syn. *Komagataella* sp.) have gained great interest as production hosts for recombinant proteins [10] and more recently also as platform for metabolite production [2]. Both applications require promoter collections of different strength for metabolic and cell engineering to enable and enhance productivity. Promoter libraries were developed based on mutating transcription factor binding sites [11], or by random mutagenesis [8]. Strong constitutive and regulated promoters were identified by transcriptomics studies [12, 13]. Delic et al. [14] described a collection of native regulated promoters of different strength with the main aim of providing repressible promoters for gene knockdown studies. Synthetic core promoters represent a source for transcriptional initiators at different strength, however with the loss of regulatory features [1, 15].

A specific feature of methylotrophic yeasts is the carbon source dependent regulation of the genes involved in methanol metabolism. Recently we have redefined the methanol assimilation pathway of *P. pastoris* [16], a finding that was initially based on the identification of all genes that are upregulated on methanol as a substrate. These include hitherto unknown genes, controlled by promoters of a wide range of expression strength on methanol (Table 1). Beside different expression levels upon induction by methanol, these promoters feature a wide variety of induction degrees, defined as the ratio of expression levels in the induced state (presence of methanol) vs. the non-induced state (cells grown on glucose or glycerol). Some of these promoters are even deregulated on substrate limit without addition of methanol, illustrating a variety of regulation patterns which can be summarized by correlating the genes according to the similarity of their regulatory behavior in a plethora of different growth conditions, such as different carbon sources [17] or different growth rates, featuring different degrees of substrate limitation [18]. Thus they are allowing controllable expression of genes depending on the needs or growth conditions of the host cells.

**Table 1:** Methanol regulated genes of *P. pastoris* as a source of regulated promoters

Ranked expression level (methanol)^a^	Short name	ORF name^b^	Co-regulation: 1 = with A/D/F; 2 = with A; 3 = with D/F; 4 = up at glucose limit^c^	Methanol induction^d^
1	*DAS1*	PP7435_Chr3-0352	1;4	Strong
2	*AOX2*	PP7435_Chr4-0863	2;4	Strong
3	*AOX1*	PP7435_Chr4-0130	1;4	Strong
4	*DAS2*	PP7435_Chr3-0350	3;4	Strong
5	*FDH1*	PP7435_Chr3-0238	1;4	Strong
6	*PMP20*	PP7435_Chr1-1351		Strong
7	*THI11*	PP7435_Chr4-0952		Weak
8	*FLD*	PP7435_Chr3-0140	3	Intermediate
9	*FBA1*-*2*	PP7435_Chr1-0639	1	Strong
10	*SHB17*	PP7435_Chr2-0185	3	Intermediate
11	*FGH1*	PP7435_Chr3-0312	1	Intermediate
12	*DAK2*	PP7435_Chr3-0343	3	Intermediate
13	*CTA1*	PP7435_Chr2-0137	3	Weak
14	*PMP47*	PP7435_Chr3-1139	1	Strong
15	*MPP1*	PP7435_Chr3-0349	3	Weak
16	*FBP1*	PP7435_Chr3-0309	3	Weak
17	*PIM1*-*2*	PP7435_Chr1-0484	2	Weak
18	PAS_chr1-1_0037	PP7435_Chr1-0336	1	Strong
19	PAS_chr3_1071	PP7435_Chr3-0094	1	Strong
20	*PEX11*	PP7435_Chr2-0790	3;4	Intermediate
21	*PEX13*	PP7435_Chr2-0217	1	Weak
22	PAS_chr1-1_0343	PAS_Chr1-1_0343	4	Intermediate
23	*PEX12*	PP7435_Chr4-0200	1	Weak
24	*INP1*	PP7435_Chr4-0597	3	Weak
25	*PEX6*	PP7435_Chr1-0900	1	Weak
26	*PEX17*	PP7435_Chr4-0347	1	Weak
27	*ATG37*	PP7435_Chr4-0369	1	Weak
28	*TAL1*-*2*	PP7435_Chr2-0358	1	Intermediate
29	*PEX5*	PP7435_Chr2-0195	3	Intermediate
30	*PEX2*	PP7435_Chr3-1201	3	Weak
31	PAS_chr3_1020	PP7435_Chr3-0149	3	Strong
32	*PEX1*	PP7435_Chr3-0122	1	Weak
33	*PEX26*	PP7435_Chr4-0482	1	Weak
34	*PEX10*	PP7435_Chr1-1379	3	Weak
35	*PEX14*	PP7435_Chr4-0157	3	Weak
36	PAS_chr3_0408	PP7435_Chr3-0805		Intermediate
37	*ARO7*	PP7435_Chr4-0965	3	Weak
38	*PEX8*	PP7435_Chr1-1134	1	Weak
39	PAS_chr1-4_0459	PP7435_Chr1-1255	1	Intermediate
40	*FAD1*	PP7435_Chr1-0246		Intermediate
41	YLR177 W	PP7435_Chr1-0659	3	Intermediate
42	*PEX11C*	PP7435_Chr1-1331	3	Weak
43	*ACS2*	PP7435_Chr3-0810		Weak
44	PAS_chr3_0439	PAS_chr3_0439	2	Intermediate
45	*RKI1*-*2*	PP7435_Chr4-0797	3	Intermediate

^a^Relative gene expression levels were derived from signal intensities on DNA microarrays at methanol induction [16, 17] and ordered from highest to lowest

^b^ORF names derived from published *P. pastoris* genome sequences [19, 20]

^c^The gene correlation was calculated using transcriptomic datasets comprising 29 different conditions. The log_2_ fold change data was used to look for co-regulations in this data set. The data was processed via the DeGNServer to calculate Spearman´s rank correlation using a CLR-based Network and an association cut-off value of 3.8 [21]. Co-regulation was analyzed with three genes involved in methanol utilization: *AOX1* (A), *DAS1* (D), *FBA1*-*2* (F). Up at glucose limit means that expression is deregulated in glucose limited culture conditions without methanol (data from [12])

^d^Induction on methanol was classified based on the transcriptional regulation patterns obtained by [16, 17] by comparing expression levels of cells grown on methanol to cells grown on glucose or glycerol

## Conclusions

Genome scale transcriptomic studies are a valuable source of information on native promoters and have been successfully used to identify promoters of different strength and desired regulatory behavior. Well defined promoters are core elements of synthetic biology part collections. The collection of *P. pastoris* promoters presented here, and others analyzed in the cited references can serve as a basis for setting up a *P. pastoris* promoter collection. Promoters with different regulatory strength are crucial elements of toolboxes for cell and metabolic engineering. In addition, they can be directly employed for gene expression to produce heterologous proteins or metabolites in yeasts.
